# Endotoxin tolerance and cross-tolerance in mast cells involves TLR4, TLR2 and FcεR1 interactions and SOCS expression: perspectives on immunomodulation in infectious and allergic diseases

**DOI:** 10.1186/1471-2334-10-240

**Published:** 2010-08-14

**Authors:** Saulo F Saturnino, Roberta O Prado, José R Cunha-Melo, Marcus V Andrade

**Affiliations:** 1 Department of Internal Medicine, School of Medicine, Federal University of Minas Gerais, Av. Prof. Alfredo Balena 190, Belo Horizonte, Minas Gerais, 30130100, Brazil; 2 Department of Surgery, School of Medicine, Federal University of Minas Gerais, Av. Prof. Alfredo Balena 190, Belo Horizonte, Minas Gerais, 30130100, Brazil

## Abstract

**Background:**

The study of the endotoxin tolerance phenomenon in light of the recently defined roles of mast cells and toll-like receptors as essential components of the innate immune response and as orchestrators of acquired immunity may reveal potentially useful mechanisms of immunomodulation of infectious and allergic inflammatory responses, such as sepsis or asthma. Here we evaluated the phenomenon of direct tolerance of endotoxins, as well as the induction of cross-tolerance and synergism by stimulation with toll-like receptor-2 (TLR2) and FcεR1 agonists, in murine mast cells prestimulated with lipopolysaccharide (LPS). Additionally, we evaluated some stimulatory and inhibitory signaling molecules potentially involved in these phenomena.

**Methods:**

MC/9 cells and primary bone marrow-derived mast cells obtained from C57BL/6 and TLR4^-/- ^knock-out mice were sensitized to DNP-HSA (antigen) by incubation with DNP-IgE and were prestimulated with LPS for 18 hr prior to stimulation. Cultures were stimulated with LPS or Pam3Cys-Ser-(Lys)4 3HCl (P3C), a TLR2 agonist, individually or in combination with antigen. The production of IL-6 and TNFα, the phosphorylation of NFκB and p38 MAPK, and the expression of TLR4 and SOCS-1 and -3 were analyzed.

**Results:**

We found that production of TNFα and IL-6 in murine mast cells that have been pretreated with LPS and challenged with TLR4 (LPS) or -2 (P3C) agonists was reduced, phenomena described as endotoxin tolerance (LPS) and cross-tolerance (P3C), respectively. The expression of TLR4 was not affected by LPS pretreatment. Our results show that the FcεR1 agonist DNP-HSA (antigen) interacts synergistically with LPS or P3C to markedly enhance production of cytokines (TNFα and IL-6). This synergistic effect with LPS and P3C was also attenuated by LPS pretreatment and was mediated by TLR4. These results may be attributed to the reduction in phosphorylation of the mitogen-activated protein kinase (MAPK), p38, and the transcription factor NFκB, as well as to an increase in the expression of the suppressors of cytokine signaling (SOCS)-1 and -3 proteins in LPS-pretreated mast cells.

**Conclusions:**

These findings can be explored with respect to the modulation of inflammatory responses associated with infectious and allergic processes in future studies.

## Background

Currently, treatment of sepsis is ineffective and few therapeutic innovations have been developed to improve it [1,2]. Since 1972, stemming from the quasi-intuitive ideas of Thomas Lewis [3], the concept that our immune response to infection is the sustaining foundation for sepsis has been widely accepted. An increased understanding of sepsis pathophysiology has provided stronger support for this hypothesis [4]. During the second half of the 1990 s, two coinciding facts led to new perspectives on a different approach to sepsis. First, the discovery of toll-like receptors (TLR) [5,6] initiated our present comprehension of how the host recognizes pathogen molecular patterns, how the innate response is initiated, and how the acquired immune response is organized [7]. Second, in 1996, Bernd Echtenacher et al. determined the essential role of mast cells in the innate immune response using a model of peritoneal sepsis in mast cell-deficient mice [8]. Additional work from other groups (reviewed in 9) elevated these cells, previously considered mere effectors, to the category of sentinels of the innate immune system and organizers of the adaptive immune response. In fact, their permanent location in sites likely to suffer invasion by pathogens - skin, paranasal sinuses, lungs, and intestinal mucosa - places them in a privileged position in terms of detection and subsequent organization of the immune response. Innumerable possibilities for modulation of the inflammatory response appeared after a better understanding of the role played by TLRs was gained, having as targets their stimulatory and inhibitory signaling pathways [10]. The accumulation of evidence defining mast cells as fundamental in the immune response to sepsis [11-13] opens up new perspectives on the mechanisms of immunomodulation by this type of cell [14].

Interaction between responses to infectious and allergic stimuli has been suggested on several levels, raising the possibility of a common pathway. Genetics-based studies have revealed the association of polymorphisms in the myosin light chain kinase (MYLK) gene with increased risk of sepsis and acute pulmonary injury. This same gene is involved in other inflammatory pathologies, including bronchial asthma [15]. Epidemiological data have demonstrated the effect of exposure to TLR agonists on the incidence of allergic phenomena [16]. Experimental studies in human mast cell cultures have shown an interaction between FcεR1 receptors and TLR2 [17]. In bone marrow derived-mast cells (BMMC), we have previously demonstrated the synergic action of co-stimulation of FcεR1 and TLR in the production of inflammatory cytokines [18].

The endotoxin tolerance phenomenon was described more than 60 years ago [19], and it is characterized by hyporesponsiveness to endotoxin exposure, induced by prior exposure. Cross-tolerance, which is defined by tolerance of a different stimulus induced by endotoxin, was described later. In the context of TLR signaling, the tolerance phenomenon can be used as a tool for identification of signaling molecules that can attenuate the inflammatory response, revealing potential participants in immunomodulation. Mast cells are some of the first cells to have contact with invading pathogens; when activated, they release immunoregulatory cytokines that organize the inflammatory response. The release of TNFα and the recruitment of circulating leukocytes are essential elements of the immune response that are attributed to mast cells, and characteristically, mast cells are the only type of cell that can store pre-formed TNFα and release it when activated [20]. Few studies have addressed endotoxin tolerance in mast cells, likely due to the fact that its primary role in response to infection has been defined so recently. In other cell types, where endotoxin tolerance have been more thoroughly evaluated, the proteins suppressors of cytokine signaling (SOCS) are involved in tolerance phenomena as negative regulators of the pro-inflammatory response, via the TLR4-NFκB pathway [21].

We tested the hypothesis that mast cells display the phenomenon of direct tolerance to endotoxins after prestimulation of BMMC with LPS, and that this could induce cross-tolerance for stimulation with FcεR1 and TLR2 agonists. The release of TNFα and IL-6 was measured, and the same experiment was conducted in BMMC obtained from TLR4^-/- ^knock-out (KO) mice to determine the role of TLR4 in the induction of cross-tolerance and in the potentiation of this response by co-stimulation. Additionally, we evaluated the effects of LPS prestimulation on phosphorylation of the mitogen-activated protein kinase, p38, and the transcription factor NFκB, as well as the expression of the SOCS-1 and -3 proteins, signaling molecules involved in cytokine production.

## Methods

### Materials

Reagents were obtained from the following sources: culture medium and reagents from Invitrogen/GIBCO (Carlsbad, CA); recombinant mouse IL-3 from Pepro Tech (Rocky Hill, NJ); IgE monoclonal anti-DNP antibody and its antigen, dinitrophenylated human serum albumin (DNP-HSA), and highly purified lipopolysaccharide (LPS, Escherichia coli 055:B5) from Sigma (St. Louis, MO); Pam3Cys-Ser-(Lys)4 3HCl (P3C) from EMC Microcollections GmbH (Tuebingen, Germany); and polyclonal antibodies that detect phospho-p38 MAPkinase (Thr180/Tyr182), phospho-NFκB (Ser536), and the proteins themselves from Cell Signaling Technology (Danvers, MA).

### Mast cell culture

Bone-marrow derived mast cells (BMMC) were cultivated in suspension in 75 cubic milliliter flasks (300,000 cell/ml) in RPMI-1640 supplemented with 5% fetal bovine serum, 30 ng/ml of IL-3, 2 mM glutamine, 100 μM non-essential amino acids, 10 μM 2-mercaptoethanol, and 1 mM sodium pyruvate. The MC/9 mast cell line was cultured in the same culture medium without IL-3. BMMC were obtained from wild-type (C57BL/6) and TLR4^-/- ^KO mice, under approval of the Ethics in Animal Experimentation Committee [Comitê de Ética em Experimentação Animal] of the Federal University of Minas Gerais (CETEA-UFMG), using previously described procedures [22], and the cells were cultivated in a CO_2 _incubator at 37 degrees Celsius. BMMC were utilized in experiments after 4 weeks in culture and showed 99% mast cells when stained with toluidine blue.

### Experimental protocols

BMMC were sensitized to DNP-HSA (antigen) with 100 ng/ml DNP-IgE and were pretreated with LPS (10 ng/ml or LPS 100 ng/ml) for 18 hours prior to stimulation. After 18 hours, cells were washed 3 times with cell culture medium, resuspended in complete medium and stimulation was performed with antigen (20 ng/ml), LPS (1 μg/ml), or P3C (1 μg/ml) alone or in combinations (antigen+LPS or antigen+P3C) for 24 hours for analyzing production of cytokines or 30 min for measuring degranulation. For signaling studies, BMMC were washed 3 times with HEPES buffer and stimulated in the same buffer for 30 minutes with antigen (20 ng/ml), LPS (1 μg/ml), or a combination of antigen+LPS.

### Assay for cytokine measurement

TNFα and IL-6 were measured in the supernatant after 24 hours of stimulation using the enzyme-linked immunosorbent assay (ELISA) method (BioSource kits, Camarillo, CA).

### Measurement of degranulation

For these measurements, cultures were washed and the medium was substituted with HEPES-buffered saline medium before stimulation. Degranulation was determined by the measurement of β-hexosaminidase marker release by a colorimetric assay in which the release of p-nitro phenol (coming from p-nitro phenyl-N-acetyl-β-D-glucosaminide) is measured. Values are expressed as the percentage of β-hexosaminidase that is released into the medium.

### Immunoblotting

Cells were lysed in a protease/phosphatase-inhibiting buffer by the addition of 100 μl of this solution to the cell suspension. This buffer consisted of Complete Protease Inhibitor Cocktail (Roche Molecular Biochemicals, Indianapolis, IN), Sigma protease inhibitor cocktail (Sigma, St Louis, MO), 3-4 dichloroisocoumarin (Roche Molecular Biochemicals), and benzamidine, as well as the phosphatase inhibitors sodium orthovanadate, sodium pyrophosphate, and sodium fluoride (Sigma). Samples were boiled for 4 minutes, and any existing debris was removed by centrifugation at 14,000 rpm for 5 minutes prior to loading the gels. Proteins were separated by NuPAGE BisTris gels (Invitrogen) and then transferred to nitrocellulose membranes for immunoblotting with the primary antibodies indicated. Visualization was by chemiluminescence, and quantification was by densitometry (Kodak Image Station 4000R).

### Statistical analysis

The results are provided as mean ± SEM and were analyzed using the Student t-test with the confidence interval established at 95%. Values were considered significant if p < 0.05. Data were stored in the Graphpad Prism 4 statistical program.

## Results

### Mast cells express endotoxin tolerance not related to the reduction of toll-like receptor-4 expression

**Figure 1:**
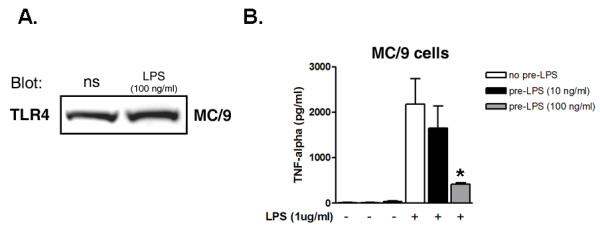
**Effect of LPS prestimulation on expression of TLR4 and on TNFα production**. The MC/9 mast cell line was prestimulated with LPS (10 and 100 ng/ml) and the expression of TLR4 (A) and the production of TNFα after a second challenge with LPS (1 μg/ml) (B) were determined by immunoblotting and ELISA, respectively. The production of TNFα was significantly reduced in a dose-dependent manner after prestimulation with LPS 100 ng/ml (* p < 0.01). However, this reduction was not mediated by a reduction in TLR4 expression, as shown. Values are mean and SEM from 3 independent experiments.

Pretreatment with 100 ng/ml of LPS for 18 hours did not affect expression of TLR4 in mast cells (Figure 1A). However, mast cells pretreated with LPS (100 ng/ml) showed lower production of TNFα upon a second challenge with LPS (1 μg/ml) compared with cells that not receive prestimulation (p < 0.05) (Figure 1B).

### LPS acts in synergy with antigens through TLR4 signaling to augment production of cytokines; this potentiation is reduced by preconditioning with LPS

**Figure 2:**
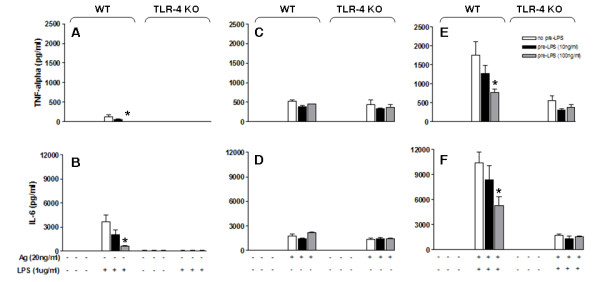
**Effect of LPS prestimulation on cytokine production after a second challenge with LPS, antigen, or a combination of both**. Reduction of TNFα (A) and IL-6 (B) production after stimulation with 1 μg/ml LPS in BMMC prestimulated with 10 ng/ml LPS (p = 0.18) and 100 ng/ml LPS (p = 0.01). No significant reduction of TNFα (C) or IL-6 (D) production was observed after stimulation with 20 ng/ml Ag in cells prestimulated with 10 ng/ml LPS (p = 0.057) and 100 ng/ml LPS (p = 0.17). Attenuation of potentiation of TNFα (E) and IL-6 (F) production by simultaneous stimulation with 20 ng/ml Ag and 1 μg/ml LPS when BMMC were prestimulated with 10 ng/ml LPS (p = 0.16) and 100 ng/ml LPS (p = 0.01). The TLR4 KO BMMC graphs (A-F) demonstrate that potentiation and attenuation with the FCεR1 agonist and LPS is mediated by TLR4 because there is no significant difference between the non-prestimulated and prestimulated groups. Of note, the synergy with the combination of LPS+Ag for TNFα (E) and for IL-6 (F) is no longer observed in TLR4 KO BMMC. Values are mean and SEM from 3 independent experiments.

Similar to the results seen with the MC/9 cell line, primary BMMC pretreated with LPS (100 ng/ml) showed lower production of TNFα upon a second challenge with LPS (1 μg/ml), (p = 0.01). Prestimulation with LPS (10 ng/ml) was non-significant (p = 0.18) (Figure 2A, WT). As for IL-6, the same was observed with lower production upon prestimulation with LPS 100 ng/ml (p = 0.01) and non-significant with 10 ng/ml (p = 0.17) (Figure 2B, WT). In comparison with BMMC that did not receive prestimulation, cells prestimulated with LPS (100 ng/ml) displayed significantly reduced production of TNFα and IL-6 upon a second challenge with LPS (1 μg/ml). These results illustrate that prior exposure to endotoxins induces a significant reduction in the production of these proinflammatory cytokines upon a second stimulation with LPS (Figures 2A and 2B, WT). In BMMC from TLR4 knockout mice, we did not detect production of TNFα or IL-6 in response to a second challenge with LPS, in cells that were either pretreated or not treated with LPS (Figure 2A and 2B, TLR4 KO).

To test the hypothesis that a common pathway may modulate responses to stimulation of the TLR4 and FcεR1 receptors, BMMC were incubated with DNP-IgE (50 ng/ml), prestimulated with LPS (10 ng/ml or 100 ng/ml) for 18 hours, and then stimulated with antigen (20 ng/ml). Prestimulation with 10 ng/ml LPS (p = 0.057) and 100 ng/ml LPS (p = 0.17) did not reduce production of TNFα when the cells were stimulated with antigen (Figure 2C, WT). As for IL-6, there was no reduction with prestimulation with LPS 10 ng/ml (p = 0.1) and 100 ng/ml (p = 0.1) (Figure 2D, WT). The lack of TLR4 did not affect the production of cytokines in response to antigen (Figures 2C and 2D, TLR4 KO).

As we have previously demonstrated [18], simultaneous stimulation of the FcεR1 and TLR4 receptors results in greater production of cytokines (TNFα and IL-6) when compared to the individual stimulation of each. In support of the common pathway hypothesis, we demonstrate two new observations in this sequence of events. First, we show the attenuation of synergy in TNFα (p = 0.01) (Figure 2E) and IL-6 (p = 0.03) (Figure 2F) production between LPS and antigen after preconditioning with LPS (100 ng/ml). Second, this synergism is dependent on TLR4, as will be demonstrated further on. Therefore, prestimulation with LPS (100 ng/ml) results in attenuation of the synergistic effect of co-stimulation with LPS and antigen.

To determine the role of TLR4, we conducted experiments using the same protocol of prestimulation with LPS, but stimulated BMMC that do not express TLR4, which were obtained from TLR4-knockout mice. Neither tolerance nor a tendency to lower production of proinflammatory cytokines were evident when analyzing the production of TNFα and IL-6 by mast cells obtained from TLR4 KO mice, whether prestimulation was performed with LPS at 10 or 100 ng/ml. The synergistic effect of simultaneous stimulation is thus mediated by TLR4 because this phenomenon was not observed in mast cells that do not express TLR4 (Figures 2E and 2F, TLR KO).

### Preconditioning with LPS did not affect the intensity of degranulation

For analysis of the effects of prestimulation with LPS (10 ng/ml or 100 ng/ml) on degranulation, we measured the percentage of β-hexosaminidase released after stimulation with antigen (20 ng/ml), LPS (1 μg/ml) or a combination of the two. There was no significant change in the percentage of degranulation in response to any of the stimuli (data not shown).

### Preconditioning with LPS determines cross-tolerance to the toll-like receptor 2 agonist P3C

**Figure 3:**
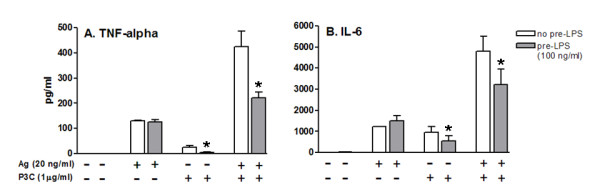
**Effect of LPS prestimulation on cytokine production after a second challenge with antigen, a TLR2 agonist (P3C), or a combination of both**. BMMC were prestimulated with LPS (100 ng/ml) for 18 hours. Next, the cells were stimulated with 20 ng/ml Ag, 1 μg/ml P3C, or a combination of both for 24 hours for analysis of the production of cytokines. Reduction of TNFα (A) and IL-6 (B) production was seen after stimulation with P3C alone (* TNFα p = 0.047; * IL-6 p = 0.029) and in combination with Ag + P3C (* TNFα p = 0.012; * IL-6 p = 0.004), but not with Ag alone (p > 0.05), suggesting a cross-tolerance effect linking TLR2 and TLR4 agonists. Values are mean and SEM from 3 independent experiments.

After analyzing the influence of TLR4 expression on tolerance phenomena in mast cells, we tried to determine a possible common pathway linking TLR4 and TLR2 responses in endotoxin tolerance. BMMC were prestimulated with LPS (100 ng/ml) for 18 hours. After 18 hours, the cells were stimulated with antigen (20 ng/ml), P3C (1 μg/ml), or a combination of both for 24 hours for analysis of the production of cytokines. Reduction of TNFα and IL-6 production after stimulation with P3C alone (TNFα p = 0.047; IL-6 p = 0.029) or with the combination of antigen + P3C (TNFα p = 0.012; IL-6 p = 0.004) was observed, but this reduction was not seen with antigen alone (Figure 3A and 3B).

### Prestimulation with LPS leads to an increase in SOCS expression and in attenuation of phosphorylation of NFκB and p38 MAPkinase after co-stimulation with antigen and LPS

**Figure 4:**
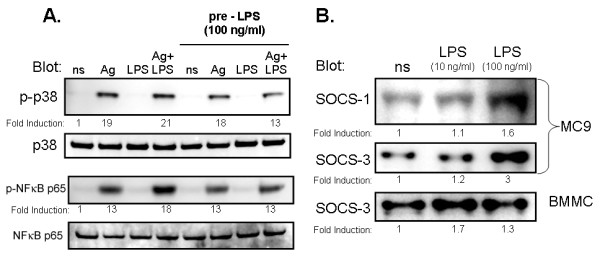
**Effects of LPS prestimulation on SOCS expression and on phosphorylation of NFκB-p65 and p38 after a second challenge with antigen, LPS, or a combination of both**. BMMC were prestimulated with 10 ng/ml LPS or 100 ng/ml LPS for 18 hours. Next, the cells were stimulated with 20 ng/ml antigen, 1 μg/ml LPS, or a combination of both for 30 min. Figure 4A: NFκB-p65 phosphorylation was increased by co-stimulation with antigen + LPS and this synergistic effect was reduced by prestimulation with LPS, probably corresponding to the reduction in TNFα and IL-6 production. Under prestimulation, p38 phosphorylation was also reduced when stimulated with the combination of Ag and LPS. Figure 4B: Prestimulation with LPS by itself resulted in increased expression of SOCS-1 and -3 in MC/9 and SOCS-3 in BMMC cells. The blots shown were typical results from three experiments. For Figure 4B, images were cropped to allow direct visual comparison from non-stimulated and stimulated cells from the same gel. Fold induction is the mean of three individual experiments from two BMMC cultures.

We also conducted experiments with BMMC that have been prestimulated with LPS (100 ng/ml) and with non-prestimulated cells as a control group. After 18 hours, pre- and non-prestimulated BMMC were stimulated with 1 μg/ml LPS, 20 ng/ml antigen, or a combination of both for 30 min. NFκB phosphorylation was increased by co-stimulation with antigen and LPS, and this synergistic effect was reduced by prestimulation with LPS. After prestimulation with LPS, p38 phosphorylation was also reduced when stimulated with the combination of antigen and LPS (Figure 4A). Prestimulation with LPS alone resulted in increased expression of SOCS-1 and -3 in MC/9 cells and increased SOCS-3 in BMMC cells (Figure 4B). We could not detect expression of SOCS-1 in BMMC cells.

## Discussion

With the increased understanding of the immune response-regulating mechanisms introduced during the second half of the 1990 s, models of tolerance to endotoxins have been seen as a tool to identify mediators and opportunities in modulation of the inflammatory response, particularly in sepsis [23]. Our results elucidate the phenomenon of direct endotoxin tolerance mediated by TLR4-NFκB pathway and cross-tolerance of TLR2 agonists in mast cells. Furthermore, we show synergism between TLR4 and FcεR1 agonists and attenuation of this response after prestimulation with LPS. These findings may result at least in part from a reduction in the phosphorylation of the mitogen-activated protein kinase, p38, and the transcription factor NFκB, as well as the increase in expression of SOCS-1 and -3 in mast cells.

Membranes of mast cells express Toll-like receptors that, under stimulation by components of pathogens, lead these cells to release cytokines, particularly TNFα and other effector molecules. These cytokines then control the behavior of other cells, promoting inflammation and afflux to the infection site [24]. The reprogramming induced by LPS in mast cells that we have shown here offers the possibility of evaluating the mechanism of modulation of the inflammatory response at its roots, both in response to first contact with the pathogen and at the level of consequent signaling events. We show that this reprogramming is not determined by reduction of TLR4 expression but is mediated by TLR4-NFκB signaling pathway. Reports of the possibility that negative regulation of the immune response might be mediated by TLR [25] may be extrapolated to address mast cells.

The synergistic interaction between TLR and FcεR1 receptors was demonstrated by our prior results [18]. The results herein confirmed this synergy and added information regarding interaction between these receptors, showing the attenuation of synergy by pre-conditioning through TLR4 stimulation with LPS. We also show the fundamental role of TLR4 in potentiation after co-stimulation, which is absent in BMMC from TLR4 KO mice. The functional correspondence to human mast cells with respect to TLR4 and FcεR1 responses was demonstrated in terms of immune response modulation, including the analysis of genetic expression [26]. Regarding sepsis, common genetic foundations define the connecting link between the risk of sepsis with acute pulmonary injury and allergic phenomena such as asthma [14]. Inflammatory response-regulating mechanisms associated with the tolerance phenomenon identified in this context can possibly be common to an infectious or allergic response.

Of note is the identification of a cross-tolerance effect linking TLR4 and TLR2 agonists, which is mediated by a factor that can influence the transduction mechanisms of both receptors in mast cells. This suggests a common signaling pathway or protein that can be a target of immunomodulation of these receptors. As these receptors are responsible for recognizing components of gram-negative (TLR4) and gram-positive (TLR2) bacteria, they have become a major target to blunt the pro-inflammatory response in sepsis. The phenomena of cross-tolerance has been previously described [27], and our observations regarding mast cells are new.

We showed attenuation of phosphorylation induced by reprogramming of mast cells after prestimulation with LPS, particularly in the TLR4-NFκB pathway (probably corresponding to the reduction in TNFα and IL-6 production observed in our experiments). These results are vital data in the search for mediators that may be used in an attempt to obtain a more balanced inflammatory response. Several negative regulators have been described as being involved in the endotoxin tolerance process, such as SH2-containing inositol phosphatase (SHIP) [28]. BMMC from SHIP knockout mice do not display endotoxin tolerance, probably acting in concert with IRAK-M and SOCS-1 to downregulate the TLR signaling pathway. In SHIP ^+/+ ^cells LPS tolerance is associated with inhibition of NF-κB activation, one of the principal endpoint of LPS signal transduction [28]. In a recent report, SOCS-1 and -3 were described as suppressor factors involved in a coordinated negative regulation of innate and adaptive immune responses in macrophages, dendritic cells and T-lymphocytes [29]. In our experiments, we show that increases in expression of SOCS-1 and -3 were associated with tolerance and cross-tolerance in mast cells. SOCS mediates a negative feedback mechanism in LPS induced TLR4 transduction, possibly acting downstream in MyD88 dependent pathway, at IRAK and p50/p65 levels, in order to reduce the production of proinflammatory cytokines [30]. The negative regulation at p50/p65 level may be involved in FcεR1/TLR4 attenuation of the synergistic activation of these receptors. Moreover, SOCS 3 inhibit ubiquitination of TRAF6, preventing association and activation of TAK1 in the TLR pathway [30]. As TRAF6 contributes to FcεR1 mediated cytokine production in mast cells [31], this mechanism is potentially relevant in further studies addressing the interaction between TLR and FcεR1 receptors. Accordingly, in mast cells, evidence has accumulated on their immunomodulating properties, presenting new opportunities to explore these cells in inflammatory response imbalance processes [32], such as sepsis or bronchial asthma.

## Conclusions

Mast cells express endotoxin tolerance, and our data demonstrate dependence on TLR4 for this phenomenon to occur in response to co-stimulation with LPS and antigen. These facts strengthen the evidence of interaction between inflammatory responses caused by infection or allergy through TLR4 and FcεR1 receptors. Moreover, evidence in the literature on the role of mast cells in innate immunity and the results presented here regarding cross-tolerance between TLR4 and TLR2 highlight the relevance of these findings with respect to sepsis. The association of endotoxin tolerance, cross-tolerance involving TLR4, TLR2, FcεR1 receptors, and SOCS expression implicate these proteins as potential modulators of innate and adaptive responses. In mast cells, recently defined as coordinators of innate and adaptive immunity, use of these potential modulators likely produces a more balanced inflammatory response in processes in which imbalance determines damage, such as in sepsis and allergic diseases.

## Competing interests

The authors declare that they have no competing interests.

## Authors' contributions

SFS was responsible for study design, acquisition of data, analysis and interpretation of data, and drafting of the manuscript. ROP performed the western blotting. JRCM analyzed and interpreted the data. MVA was responsible for study design, acquisition of data, the analysis and interpretation of data and drafting of the manuscript. All authors have read and approved the final manuscript.

## Pre-publication history

The pre-publication history for this paper can be accessed here:

http://www.biomedcentral.com/1471-2334/10/240/prepub
